# High Prevalence of Hypertension and Placental Insufficiency, but No *In Utero* HIV Transmission, among Women on HAART with Stillbirths in Botswana

**DOI:** 10.1371/journal.pone.0031580

**Published:** 2012-02-22

**Authors:** Roger L. Shapiro, Sajini Souda, Natasha Parekh, Kelebogile Binda, Mukendi Kayembe, Shahin Lockman, Petr Svab, Orphinah Babitseng, Kathleen Powis, William Jimbo, Tracy Creek, Joseph Makhema, Max Essex, Drucilla J. Roberts

**Affiliations:** 1 Division of Infectious Diseases, Beth Israel Deaconess Medical Center, Boston, Massachusetts, United States of America; 2 Department of Immunology and Infectious Diseases, Harvard School of Public Health, Boston, Massachusetts, United States of America; 3 The Botswana–Harvard School of Public Health AIDS Institute Partnership for HIV Research and Education, Gaborone, Botswana; 4 Botswana National Health Laboratory, Ministry of Health, Gaborone, Botswana; 5 Infectious Disease Unit, Brigham and Women's Hospital, Boston, Massachusetts, United States of America; 6 Princess Marina Hospital, Ministry of Health, Gaborone, Botswana; 7 Departments of Medicine and Pediatrics, Massachusetts General Hospital, Boston, Massachusetts, United States of America; 8 Centers for Disease Control and Prevention, Atlanta, Georgia, United States of America; 9 Department of Pathology, Massachusetts General Hospital, Boston, Massachusetts, United States of America; Vanderbilt University, United States of America

## Abstract

**Background:**

Increased stillbirth rates occur among HIV-infected women, but no studies have evaluated the pathological basis for this increase, or whether highly active antiretroviral therapy (HAART) influences the etiology of stillbirths. It is also unknown whether HIV infection of the fetus is associated with stillbirth.

**Methods:**

HIV-infected women and a comparator group of HIV-uninfected women who delivered stillbirths were enrolled at the largest referral hospital in Botswana between January and November 2010. Obstetrical records, including antiretroviral use in pregnancy, were extracted at enrollment. Verbal autopsies; maternal HIV, CD4 and HIV RNA testing; stillbirth HIV PCR testing; and placental pathology (blinded to HIV and treatment status) were performed.

**Results:**

Ninety-nine stillbirths were evaluated, including 62 from HIV-infected women (34% on HAART from conception, 8% on HAART started in pregnancy, 23% on zidovudine started in pregnancy, and 35% on no antiretrovirals) and 37 from a comparator group of HIV-uninfected women. Only 2 (3.7%) of 53 tested stillbirths from HIV-infected women were HIV PCR positive, and both were born to women not receiving HAART. Placental insufficiency associated with hypertension accounted for most stillbirths. Placental findings consistent with chronic hypertension were common among HIV-infected women who received HAART and among HIV-uninfected women (65% vs. 54%, p = 0.37), but less common among HIV-infected women not receiving HAART (28%, p = 0.003 vs. women on HAART).

**Conclusions:**

*In utero* HIV infection was rarely associated with stillbirths, and did not occur among women receiving HAART. Hypertension and placental insufficiency were associated with most stillbirths in this tertiary care setting.

## Introduction

Increased stillbirth rates occur among HIV-infected women, but no studies have evaluated the pathological basis for this increase, or whether highly active antiretroviral therapy (HAART) influences the etiology of stillbirths. It is also unknown whether HIV infection of the fetus is associated with stillbirth.

Several studies from the pre-HAART era demonstrate an increase in stillbirth among HIV-infected women [1]–[3], including a 1998 meta-analysis that demonstrated an odds ratio of 3.91 (95% CI 2.65–5.77) for stillbirth among HIV-infected women [4]. In some studies, women with lower CD4 cell count have had a higher stillbirth risk [1], [3], [5]. In the HAART era, national surveillance in the United Kingdom found that women receiving HAART may have had a higher risk for stillbirth than those taking one or two antiretrovirals (AOR 2.27, 95% CI, 0.96–5.41) [6]. However, other studies have not demonstrated excess stillbirth risk with HAART [7]–[9]. Only one previous study, from the pre-HAART era, has evaluated the HIV status of stillbirths to determine whether acute fetal HIV infection is associated with stillbirth events [3].

These previous studies suggest a complex relationship between HIV, HAART, and stillbirth that requires further evaluation of causal mechanisms. Few studies have performed pathologic placental examinations among HIV-infected women [10]–[15], and none to our knowledge have focused specifically on stillbirth, or the relationship between HAART and stillbirth. Therefore, we sought to identify the causes of stillbirth among a selected cohort of HIV infected and HIV uninfected women through HIV DNA PCR testing of stillbirths, pathologic examination of placentas, and detailed verbal autopsies.

## Methods

Between January 26 and November 26, 2010, we identified women who had delivered stillbirths at Princess Marina Hospital (PMH) in Gaborone, Botswana. PMH is the largest hospital in Botswana, and serves as a general delivery site as well as a referral center for high-risk deliveries in southern Botswana (which is a non-malarial region). The Health Research Development Committee from Botswana and the Harvard School of Public Health Office of Human Research Administration approved the study protocol. Written informed consent was obtained from all participants.

We asked women who experienced stillbirth at >19 weeks gestation (as determined by available obstetric records) to enroll in the study if they were willing to provide informed consent, had a placenta saved for potential examination, and agreed to additional laboratory testing. The target sample size was 100 women, and to enrich the cohort for HIV-infected women, we limited the enrollment of HIV-uninfected women to a maximum of 40 women. All HIV-infected women who delivered stillbirths were sought for potential enrollment, whether receiving HAART, ZDV, or no antiretrovirals. All antiretrovirals were provided by the Botswana government to its citizens according to national guidelines (non-citizens were required to purchase the recommended antiretrovirals). HAART was offered for maternal treatment, either before or during pregnancy, for CD4 cell counts <250 cells/mm^3^ or an AIDS-defining illness. ZDV was offered to those who did not qualify for treatment, starting at 28 weeks of pregnancy. Nadir CD4 cell counts were available for only a small number of women from the medical records and could not be analyzed. Pre-HAART HIV RNA testing is not routinely performed in the Botswana treatment program.

Abstracted obstetrical records were used to obtain details of the pregnancy, maternal medical history, antiretroviral receipt in pregnancy (for HIV-infected women), and information about the delivery. A maternal questionnaire and verbal autopsy tool was used to confirm data from the obstetrical record and to identify additional information about the pregnancy and delivery. Women with unknown HIV status, and women who tested HIV negative >12 weeks before delivery, were offered HIV testing by rapid ELISA. Among HIV-infected women, HIV RNA testing (COBAS Amplicor HIV-1 Monitor Test v.1.5, Roche Molecular Systems Inc., New Jersey, USA) and CD4 cell count testing were performed, and HIV DNA PCR testing of blood obtained by cardiac puncture was performed for the stillborn fetus (qualitative DNA Amplicor HIV-1 test, Roche Diagnostic Systems, New Jersey, USA). All stillbirths were weighed and examined by a study physician for congenital abnormalities.

### Placental Pathology

The placentas were examined fresh using a standard template for gross descriptions and sampling. The placentas were grossly examined, weighed, and trimmed of cord and membranes. The placentas were sampled making duplicate blocks of proximal and distal umbilical cord, full thickness parenchyma, and any lesions grossly appreciated. The samples were fixed in formalin and processed at the Botswana National Health Laboratory using standard protocols into paraffin blocks. The blocks were divided and one set was transported to the Massachusetts General Hospital in Boston for routine 5 µm sectioning and H&E staining.

The placental pathology was interpreted by one pathologist (DJR) who had available the gross examination findings, gestational age as determined at delivery, fetal weight and gross findings (e.g. maceration, edema, gross malformations, etc.). The pathologist was blinded to all other clinical data including maternal and fetal HIV status, treatment, maternal blood pressure and evidence of infection. The histology was evaluated per usual standards for an experienced placental/perinatal pathologist and a diagnostic findings score sheet was completed for each case. Placental pathology diagnostic criteria were as follows: *Acute placental insufficiency* was defined as evidence of acute abruption, or two of the following: indenting, adherent, retroplacental hematoma intravillous hemorrhage, absence of chronic hypertensive findings, or marked increase in immature red cells circulating in fetal vessels. *Chronic placental insufficiency* was defined as two of the following: fetal/placental weight ratio >8, chronic abruption (retroplacental hematoma indenting and infarcting the parenchyma), placental infarcts, decidual vasculopathy, increased and enlarged syncytial trophoblastic knots (>1 knot per 3 villi or >10 nuclei per knot or knots larger in size than villi), or cord diameter <1 cm. *Infection* was defined as any of the following findings: acute chorioamnionitis with fetal vascular involvement, chorionic abscesses, diffuse chronic or acute villitis. Stillbirths were categorized as *unknown* if there were no significant placental pathologic findings that met the above definitions.

The target sample size was chosen to balance the feasibility and cost of performing specialized pathologic testing of placentas and fetal HIV PCR testing, while allowing for 80% power (with an alpha level of 0.05) to detect true differences of at least 30% for most comparisons. Differences of smaller magnitude were considered less likely to have public health significance. Data were analyzed in SAS (v.9.1). Associations with pathologic diagnoses were performed using chi square testing or by univariate logistic regression. Characteristics were compared between groups by chi square testing, or by rank-based Spearman or Wilcoxon testing.

## Results

In total, 6,192 women delivered at PMH during the study period, and 29% were HIV-infected. There were 251 stillbirths; 105 (42%) were among HIV-infected women, 119 (47%) among HIV-uninfected women, and 27 (11%) among women with unknown HIV status. We enrolled 99 women with a confirmed HIV status (accounting for 40% of all women experiencing stillbirth during the study period). Demographic data for HIV-infected and HIV-uninfected women included in the study did not differ substantially from surveillance data collected among all women who delivered stillbirths in the study period (data not shown). Our cohort was enriched for HIV infection, and included 62 (62%) who were HIV-infected, and 37 (37%) who were HIV-uninfected. Maternal characteristics by HIV status are shown in Table 1. Among HIV-infected women, maternal demographics were similar for those on or off HAART, except only 1 non-citizen received HAART in pregnancy (p = 0.04).

**Table 1:** Characteristics of Women Delivering Stillbirths, by HIV Status, Botswana.

Maternal Characteristic	HIV-infected women		HIV-uninfected women (N = 37)
	Receiving HAART (N = 26)	Not Receiving HAART (N = 36)	
Age (median)	32 yrs¶	30 yrs¶	27 yrs¶
Botswana citizen	25 (96%)§	28 (78%)§	33 (89%)
Education			
Primary	5 (19%)	7 (19%)	4 (11%)
Secondary	18 (69%)	26 (72%)	25 (68%)
Tertiary	3 (12%)	2 (6%)	6 (16%)
None/unknown	0 (0%)	1 (3%)	2 (5%)
Salaried employment	17 (65)%	14 (38%)	22 (59%)
Referred to PMH for complicated pregnancy or delivery	22 (85%)	34 (94%)	29 (78%)
Primigravidus	2 (8%)	7 (19%)	12 (32%)
Nulliparous	4 (15%)¶	8 (22%)¶	14 (38%)¶
Past stillbirth	5 (19%)	2 (6%)	5 (14%)
Hypertension in pregnancy*	14 (54%)	17 (47%)	19 (51%)
Preeclampsia/eclampsia in pregnancy**	6 (23%)	7 (19%)	8 (22%)
Antibiotics in pregnancy	14 (54%)	15 (42%)	13 (35%)
Median hemoglobin in pregnancy	11.3 g/dL	11.6 g/dL	11.1 g/dL

*Measured systolic blood pressure ≥140 mm Hg or diastolic blood pressure ≥90 mm Hg, or diagnosed with hypertension in pregnancy prior to the onset of labor.

**Presence of hypertension, proteinuria, edema, +/− seizure.

§ P-value <0.05 for difference between women receiving HAART vs. not receiving HAART.

¶ P-value <0.05 for difference between HIV-uninfected women vs. all HIV-infected women.

Among the 62 HIV-infected women, 26 (42%) were receiving HAART at the time of the stillbirth (81% from prior to conception, and 19% started during pregnancy); 14 (23%) were receiving zidovudine (ZDV) alone from a median gestation of 28 weeks, and 22 (35%) had not received antiretrovirals. A nevirapine (NVP)-based regimen was reported for 24 (92%) of 26 who received HAART. The median CD4 cell count at the time of the stillbirth delivery was 377 (range 104, 1953) cells/mm^3^ for those receiving HAART and 353 (range 44, 937) cells/mm^3^ for those not receiving HAART (p = 0.93). At delivery, median HIV RNA was <400 (range <400 to 22,339) copies/mL for women on HAART, and 8,630 (range <400, 714,000) copies/mL for women not receiving HAART (p<0.001).

Delivery characteristics and stillbirth characteristics are shown in Table 2. The median gestational age at the time of stillbirth delivery was 31 weeks for both HIV-infected and HIV-uninfected women. For HIV-infected women, delivery characteristics were similar by HAART status. Median gestational age at delivery was 30 weeks for those on HAART and 32 weeks for those not on HAART (p = 0.23). Among the 62 HIV-infected women, 53 stillbirths (85%) had successful cardiac puncture for HIV PCR testing, and only 2 (3.7%) were HIV-infected. The first of these stillbirths occurred at 32 weeks' gestation, in a woman with HIV RNA of 31,400 copies/mL and CD4 cell count of 511 cells/mm^3^, receiving ZDV for the previous 5 weeks. The second occurred at 31 weeks' gestation, in a woman with HIV RNA of 144,000 copies/mL and CD4 cell count of 44 cells/mm^3^, receiving no antiretrovirals in pregnancy. Both had placental pathology indicative of infection (necrotizing funisitis). Neither woman had a reason identified for the stillbirth at the time of the event.

**Table 2:** Characteristics of Deliveries, Stillbirths, and Placentas, by HIV Status, Botswana.

Delivery/Stillbirth Characteristic	HIV-infected women		HIV-uninfected women (N = 37)
	Receiving HAART (N = 26)	Not Receiving HAART (N = 36)	
Median gestation at delivery (range)	30 (24, 42) weeks	32 (24, 42) weeks	31 (22, 37) weeks
% delivered at term (>/ = 37 weeks)	3 (12%)	8 (22%)	2 (5%)
Vaginal Delivery	25 (96%)	35 (97%)	36 (97)%
Fetal heart heard on admission	7 (27%)	4 (11%)	9 (24%)
Median trimmed weight of placenta (range)	170 (80, 515) g§	248 (75, 845) g§	230 (100, 930) g
Median weight of stillbirth (range)	885 (460, 2640) g§	1345 (710, 3540) g§	1170 (540, 3600) g
Macerated stillbirth	18 (69%)	26 (72%)	23 (62%)
Male gender	15 (58%)	19 (53%)	18 (49%)
Congenital abnormalities present	1 (4%)¶	0 (0%)¶	5 (14%)¶
HIV-infected stillbirth by DNA PCR	0 (0%)	2 (3.7%)	–

§ P-value <0.05 for difference between women receiving HAART vs. not receiving HAART.

¶ P-value <0.05 for difference between HIV-uninfected women vs. all HIV-infected women.

**Figure 1:**
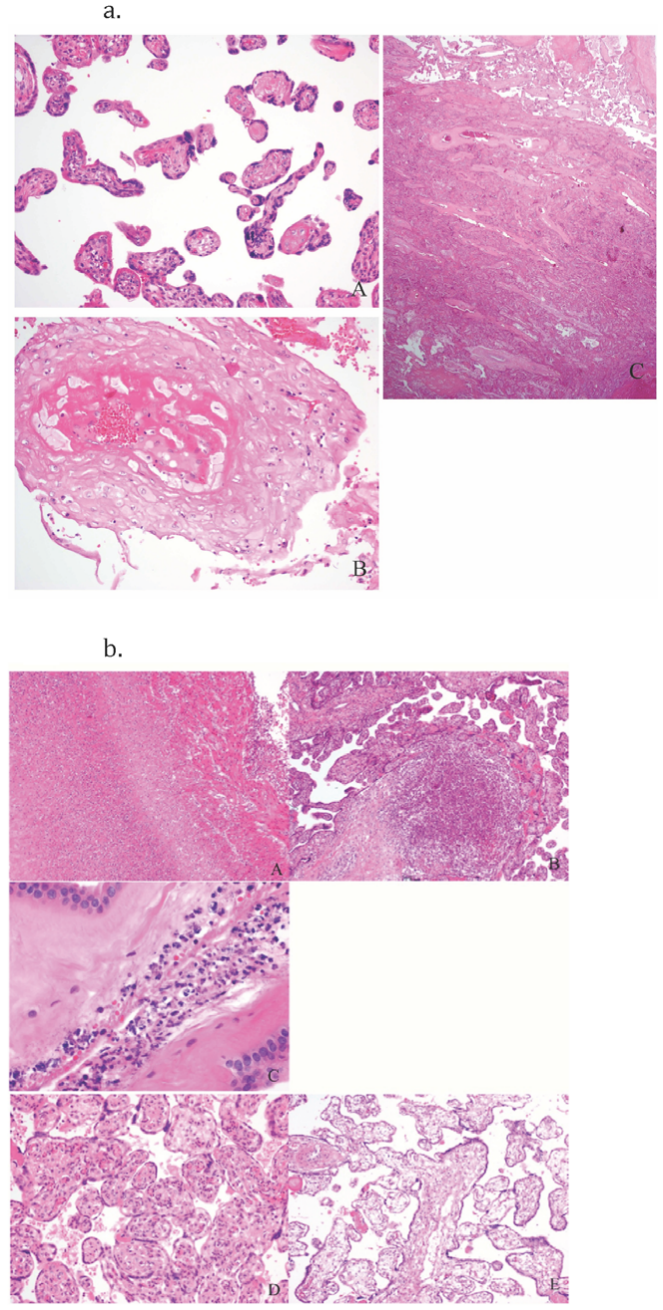
**Figure 1a** and **Figure 1b**. Figure 1a. H&E stains of placentas with typical characteristics of hypertension, Botswana. A. Distal villous hyperplasia – small round and elongate villi with large syncytial trophoblastic knots and abundant intervillous space. B. Severe decidual vasculopathy with atherosis. C.Chronic abruption. Figure 1b. H&E stains of placentas with infection and other non-hypertensive findings: A,B, and C are examples of findings that support and infectious cause of death: A. Necrotizing Funisitis – umbilical vein with transmural inflammation and necrotic neutrophil debri as a halo in Wharton's jelly. B. Acute villitis/microscopic abcess. C. Acute chorioamnionitis with multiple bacterial cocci present. D and E are other non-infectious findings: D. Villous maturational arrest (a term placenta with immature villi and centralized vessels) E. Hydrops placentalis – this placenta weighed >900 grams and showed diffuse acute villous edema.

Placental pathology results are shown in Table 3, by maternal HIV status and antiretroviral exposure status. Placental insufficiency, with features strongly suggestive of chronic placental hypertensive damage, accounted for more than half of all stillbirths in both HIV infected and HIV-uninfected women. Among women with these pathologic findings, 11 were acute, 43 were chronic, and 4 were both acute and chronic. Figure 1a demonstrates a placenta with the typical features of acute and chronic hypertension. Of women with evidence of placental insufficiency, 71% had evidence of peripheral hypertension prior to delivery, as indicated by a blood pressure measurement ≥140 mm Hg systolic or ≥90 mm Hg diastolic, or by a recorded diagnosis of hypertension during pregnancy. Of these, 73% were noted to have started an antihypertensive agent prior to delivery. Only 2 women with placental insufficiency had no recorded blood pressure or hypertension diagnosis in pregnancy. Preeclampsia (with documented proteinuria and edema) was reported in 18% of women, and eclampsia in 3%, and these did not differ by HIV status. Compared with all other women, those with placental insufficiency were more likely to report headache in pregnancy (45% vs. 17%, p = 0.003), to report puffy face in pregnancy (30% vs. 8%, p = 0.008), to require an induction of labor for intrauterine fetal demise (57% vs. 21%, p = 0.0005), and to report smaller than normal size of the stillborn (80% vs. 60%, p = 0.03).

**Table 3:** Placental Pathology Results, by HIV Status and ARV Exposure, Botswana.

Placental Pathology	HIV+ HAART in pregnancy (N = 26)	HIV+ ZDV in pregnancy (N = 14)	HIV+ No ARVs in pregnancy (N = 22)	HIV- (N = 37)
Chronic placental insufficiency	17 (65%)§	6 (43%)§	4 (18%)§	20 (54%)
Acute placental insufficiency*	1 (4%)	1 (7%)	6 (27%)	3 (8%)
Infection	4 (15%)	3 (21%)	6 (27%)	8 (22%)
Other§	2 (8%)	2 (14%)	0 (0%)	3 (8%)
Unknown	2 (8%)	2 (14%)	6 (27%)	3 (8%)

*4 women with both acute and chronic placental insufficiency were categorized as chronic.

§ Other = maternal floor infarct (2), hydrops fetalis (2), villous maturational arrest (1), massive chronic intervillositis/villitis of unknown/unclear etiology (1), massive perivillous fibrin (1).

**Figure 2:**
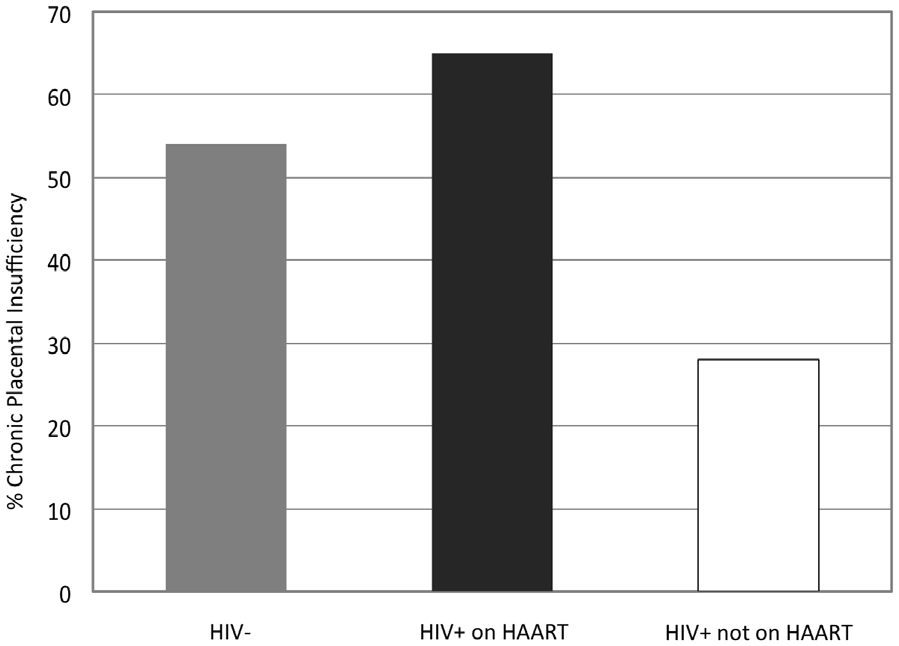
Percentage of stillbirths caused by chronic placental insufficiency, by HIV status and HAART exposure, Botswana.

Among HIV-infected women, placental insufficiency was more common among women who had received HAART than among those who had not received HAART (65% vs. 28%, p = 0.003) (Figure 2). Placental findings suggestive of acute hypertensive changes were not associated with HAART use; only 1 HIV-infected women with such findings received HAART. All but one women receiving HAART had a suppressed HIV RNA <400 copies/mL at delivery, so this variable could not be analyzed separately from HAART use itself. Similarly, only 5 women started HAART during pregnancy, so comparisons between HAART from conception vs. starting in pregnancy were limited. CD4 cell count was not associated with either acute or chronic placental insufficiency; however, median CD4 cell count at delivery was >350 cells/mm^3^ in both HAART-treated and untreated women.

Among HIV-uninfected women, the percentage with placental insufficiency with features suggestive of chronic hypertensive damage was similar to the HAART-treated HIV-infected women (54% vs. 65%, p = 0.37), and there were few women with acute placental insufficiency (8%). As with the HAART treated women, placental insufficiency was significantly more common than among HIV-infected women who were *not* receiving HAART (54% vs. 28%, p = 0.02) (Figure 2). Female stillbirth gender was noted in 70% of HIV-uninfected women with placental insufficiency (p = 0.005), although gender was equally balanced in HIV-infected women with placental insufficiency (49% female, p = 0.54).

Pathologic evidence of infection was identified in 21% of placentas from HIV-infected women, and in 22% of placentas from HIV-uninfected women (p = 0.94). Among HIV-infected women, CD4 cell count, HIV RNA, or HAART use were not associated with placental infection. Fewer maternal delivery complications were reported among women with infection compared with other women (9% vs. 53%, p = 0.0003), and more women with infection had ≥24 hours between membrane rupture and delivery (29% vs. 2%, p = 0.0001). Figure 1b shows placental pathology typical for infection and other non-hypertensive causes. There were no placentas with evidence of cytomegalovirus (CMV) infection. Necrotizing funisitis was only seen in the two stillbirths that were HIV-infected by PCR testing.

Pathologic fetal findings and congenital abnormalities were more common among stillbirths of HIV-uninfected women (14% vs. 2%, p = 0.02), and abnormalities included hydrops fetalis (1); hydrops fetalis with limb abnormalities (1); hydrops fetalis with hydrocephalus and multiple abnormalities (1); hydrocephalus (1); genito-urinary malformation (1); and rectal prolapse (1). Nuchal cord was described in 13% of deliveries, but placental pathology suggested an alternate underlying cause of the stillbirth for all but 2 of these deliveries.

## Discussion

We performed HIV PCR stillbirth testing and placental pathology in a large cohort of HIV-infected women delivering stillbirths at a referral hospital in Botswana. *In utero* HIV infection was rarely associated with stillbirths, and did not occur among those receiving HAART. Chronic placental insufficiency with features of hypertensive damage to the placenta was associated with the majority of stillbirths among HAART-treated women, and was also common among a comparator group of HIV-uninfected women. However, HIV-infected women not receiving HAART had lower rates of placental insufficiency.

Only one previous study has evaluated *in utero* HIV infection among stillbirths or miscarriages, finding 7 of 14 infections by *in situ* hybridization [3]. This pre-HAART era study was performed on women without any antiretroviral treatment in pregnancy, and HIV-infected stillbirths were associated with more advanced maternal immunosuppression. Of importance, 4 of the 7 transmissions in that study occurred at <20 weeks gestation; our study did not evaluate fetal losses at these early gestational ages. In our cohort, we report far lower vertical HIV transmission, even among untreated or ZDV-only treated women (1/22 and 1/14, respectively). Transmission did not exceed the expected amount of *in utero* MTCT for a cohort of live infants. It is unsurprising that no transmission occurred among women receiving HAART, as would be common for a series of this size evaluating live infants. In sum, our HIV DNA PCR results suggest that transmission to stillbirths probably follows a similar pattern as for live infants. Placental evaluation from both transmitting women showed findings consistent with an infectious cause of the stillbirths, with severe acute chorioamnionitis with fetal involvement, including the only cases in the series with necrotizing funisitis. We suspect, but cannot prove, that acute *in utero* HIV infection was the source of these findings and precipitated these two stillbirths.

The frequency of chronic placental insufficiency as the likely cause of stillbirths among both HIV-infected and HIV-uninfected women was surprising, as infectious etiologies are more commonly reported in low and middle income countries [16]. Although most women with chronic placental insufficiency had received a diagnosis of hypertension during pregnancy and had started an antihypertensive agent at some point during pregnancy, hypertension was diagnosed near delivery in many cases. However, we do not have complete information about treatment duration, and a more detailed review of hypertension management in Botswana is warranted. More than a quarter of women with chronic placental insufficiency had no suggestion of hypertension in pregnancy by recorded blood pressure readings or obstetrical diagnoses. While this may represent subclinical hypertension (which would add substantially to the challenge of diagnosing and managing this problem), a more likely explanation is lack of detection and the need for more aggressive hypertensive monitoring in pregnancy. Placental findings did not differ among women with or without hypertension detected during pregnancy.

HAART appeared to restore – or possibly exacerbate – the underlying risk for placental insufficiency among HIV-infected women, and those not receiving HAART had a lower risk for this complication. This finding is consistent with studies of preeclampsia and fetal death among HAART-exposed and HAART–unexposed women in European studies. In Spain, preeclampsia and fetal death rates were very low among HIV-infected women in the pre-HAART era, but jumped above background rates during the HAART era [17]. In the United Kingdom, preeclampsia was not reported among a cohort of HIV-infected women who did not receive HAART, but increased to a rate similar to HIV-uninfected controls among those receiving HAART [18]. Our findings are consistent with this literature, and suggest a hypertensive spectrum associated with HAART use that ranges from overt preeclampsia to unrecognized hypertension leading to stillbirths. A proposed mechanism for this “restoration” of risk is a shift in cytokine environment or heightened fetal antigen recognition with immune reconstitution [18], although data to support this hypothesis are lacking. The fact that most women in our study had been receiving HAART from before conception argues against an acute reconstitution event as the driving risk factor. However, few women in our study started HAART in pregnancy, and our study design could not evaluate stillbirth incidence by duration of HAART exposure. Finally, it remains unclear whether the risk of stillbirth from placental insufficiency exceeds the risk in the general population, or whether HAART merely restores the background risk. The high overall proportion of HIV-infected women delivering stillbirths at PMH during the study period (42%) may argue for the former.

Strengths of this study were the large sample size and the high percentage of total stillbirths included during the study period; the inclusion of an HIV-uninfected comparator group; and the availability of detailed verbal autopsy and medical record data, laboratory testing (including DNA PCR testing), and placental pathology. Limitations included lack of ultrasound dating for gestational ages, self-report of several factors, and limited blood pressure measurements and antihypertensive treatment histories during pregnancy. We could not distinguish potential effects of different HAART regimens, because most received NVP-based HAART. Maternal treatment regimens were linked to disease status, and although CD4 cell counts were similar for HAART-treated and non-treated women, we cannot exclude underlying disease severity as a potential confounder of stillbirth etiology (HAART-treated women were likely to have had a CD4 <250 cells/mm^3^ or AIDS-defining illness at HAART initiation, but this could not be documented from available records).

We believe that stillbirths at PMH not included in this study were the result of logistics (rapid discharge or burial) and the intentional limitation of HIV-uninfected women, and did not represent unrecognized systematic bias. We did not detect differences in key indicators between women in our cohort and all women who delivered stillbirths at PMH in the same time period. PMH is the largest general maternity ward in the country, and it is also a referral center for high-risk deliveries that may end in stillbirths, as was reported for the majority of this cohort. Although referral bias is a possibility, we estimate that PMH delivers up to half of all stillbirths that occur in hospitals in southern Botswana (BHP, unpublished data, 2011), and we believe this cohort is representative of stillbirths in the region. However, stillbirth etiologies may differ in other settings. Finally, although we strongly suspect that pathologic findings associated with the stillbirths in this study were causative (a belief strengthened by our rigorous criteria for characterizing placental findings, and the severe pathology associated with most events), we recognize that even with “gold standard” pathologic testing we cannot prove causation. Although placental features similar to chronic hypertensive damage may be detected among women with thrombophilia [19], our definitions required a constellation of placental findings only likely from chronic hypertensive damage.

In summary, we found *in utero* HIV infection was rarely associated with stillbirths, but detected a high prevalence of hypertension and chronic placental insufficiency among HAART-exposed women and HIV-uninfected women. Although HAART may reduce stillbirth risk potentially related to *in utero* HIV transmission or poor maternal health in pre-HAART era studies, it appears to restore or possibly increase risk related to placental insufficiency. However, it is unknown whether antihypertensive treatment in this setting modifies stillbirth risk, and it is likely that the severity of hypertension is an important factor in fetal outcomes [20]–[23]. A better understating of the relationship between HAART use and hypertension in pregnancy, and the mechanism by which HAART may affect chronic placental hypertensive damage, is needed. In the meantime, because hypertension in pregnancy is treatable and of potential benefit to maternal and fetal well-being, efforts to optimize screening and management of this condition for women receiving HAART are warranted in Botswana and elsewhere.
